# The Ferroelectric–Ferroelastic Debate about Metal Halide Perovskites

**DOI:** 10.1021/acs.jpclett.2c01945

**Published:** 2022-08-15

**Authors:** Francesco Ambrosio, Filippo De Angelis, Alejandro R. Goñi

**Affiliations:** † Computational Laboratory for Hybrid/Organic Photovoltaics (CLHYO), Istituto CNR di Scienze e Tecnologie Chimiche “Giulio Natta” (CNR-SCITEC), Via Elce di Sotto 8, 06123 Perugia, Italy; ‡ Department of Chemistry and Biology “A. Zambelli”, University of Salerno, Via Giovanni Paolo II 132, 84084 Fisciano, Salerno Italy; ¶ Center for Nano Science and Technology @Polimi, Istituto Italiano di Tecnologia, via G. Pascoli 70/3, 20133 Milano, Italy; § Department of Chemistry, Biology and Biotechnology, University of Perugia and UdR INSTM of Perugia, via Elce di Sotto 8, 06123 Perugia, Italy; ∥ Department of Natural Sciences & Mathematics, College of Sciences & Human Studies, Prince Mohammad Bin Fahd University, Al Khobar 31952, Saudi Arabia; ⊥ Institut de Ciència de Materials de Barcelona, ICMAB-CSIC, Campus UAB, 08193 Bellaterra, Spain; # ICREA, Passeig Lluís Companys 23, 08010 Barcelona, Spain

## Abstract

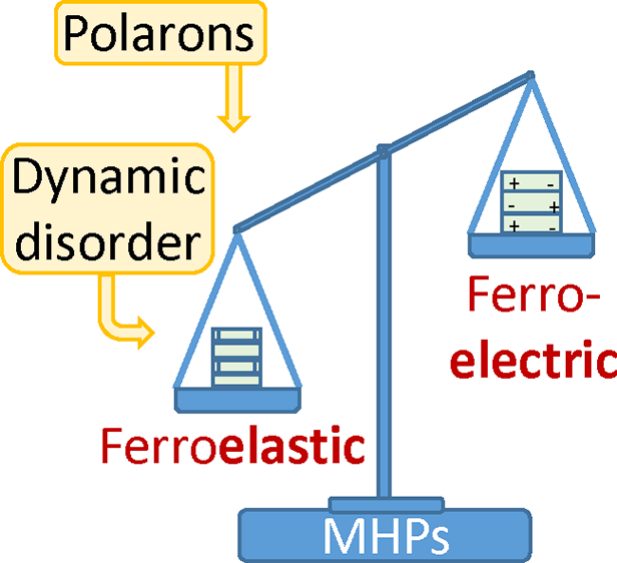

Metal halide perovskites (MHPs) are solution-processed materials with exceptional photoconversion efficiencies that have brought a paradigm shift in photovoltaics. The nature of the peculiar optoelectronic properties underlying such astounding performance is still controversial. The existence of ferroelectricity in MHPs and its alleged impact on photovoltaic activity have fueled an intense debate, in which unanimous consensus is still far from being reached. Here we critically review recent experimental and theoretical results with a two-fold objective: we argue that the occurrence of ferroelectric domains is incompatible with the A-site cation dynamics in MHPs and propose an alternative interpretation of the experiments based on the concept of ferroelasticity. We further underline that ferroic behavior in MHPs would not be relevant at room temperature or higher for the physics of photogenerated charge carriers, since it would be overshadowed by competing effects like polaron formation and ion migration.

Since the eruption of metal halide perovskites (MHPs) in the field of photovoltaics (PV) about a decade ago,^1^ these semiconductor materials have been the focus of attention worldwide. They triggered an intense, sometimes frantic, research activity, among scientists striving for a steady increase in energy conversion efficiencies, which have recently reached values beyond 25%.^2^ MHPs are really attractive because they are produced and processed by scalable, low-cost, energy-saving solution-based methods like organic semiconductors but they exhibit optoelectronic properties rivaling those of their inorganic counterparts. In fact, MHPs feature strong band-edge absorption^3^ above 1 × 10^5^ cm^–1^, small exciton binding energies^4^ around 15 meV, extremely large charge-carrier diffusion lengths^5−7^ of several tens of microns, low electron–hole recombination rates^8,9^ in the range 5 × 10^–10^ cm^3^ s^–1^, and, consequently, very long carrier lifetimes^10^ on the μs scale. The exceptional optoelectronic properties of MHPs are tightly linked to structural peculiarities. MHPs pertain to the class of materials with the chemical formula AMX_3_, where A is a positively charged cation, M is a metal, usually Pb or Sn, and X is a halide (Cl, Br, or I) anion. In hybrid perovskites, the A-site cation is an organic molecule like methylammonium (MA) or formamidinium (FA), while in fully inorganic materials, the A site is usually occupied by a Cs^+^ ion. The crystal structure is characterized by a soft and labile network of corner-sharing metal halide octahedrons (MX_6_), which at room or higher temperature usually adopts a cubic structure, reducing its symmetry to tetragonal and orthorhombic with decreasing temperature. Depending on temperature and thus on crystal structure, the A-site cations can be partly bound or almost freely moving inside the cages formed by the inorganic framework. As a consequence, MHPs exhibit pronounced lattice anharmonicity, in which the interplay between the inorganic cage and A-site cation dynamics plays an important role.^11−13^ In addition, the presence of Pb lone pairs might lead to lattice instabilities that trigger the breaking of inversion symmetry, allowing the appearance of permanent electric dipoles.^14^ This feature, coupled with the recurrent observation of well-defined domains mainly in scanning probe microscopy images, is certainly at the origin of the intense debate about the existence or absence of ferroelectricity in MHPs.

The aim of this Perspective is two-fold: On one hand, we would like to settle the controversy about ferroelectricity by exposing the main experimental and theoretical facts and revising their interpretation from the alternative point of view of ferroelasticity. In particular, we aim to convey the message that a ferroelectric polarization cannot be sustained when the A-site cation dynamics is fully unleashed, in frank contrast to the formation of ferroelastic domains. On the other hand, we will discuss the possible impact of the ferroic behavior of MHPs on the charge-carrier generation and transport, taking into account the magnitude of the observed effects in comparison with competing ones like polaron formation and ion migration.

## Is Ferroelectricity Key to the Performance of MHPs?

We consider first the previous question about the possible impact of ferroelectricity on the extraordinary performance of MHPs, particularly under operative conditions in solar cells. Once this issue is clarified, we will approach the ferroelectric–ferroelastic conundrum by listing pros and cons of both ferroic models, to finally carry out a comparative analysis aimed at reconciling theory and experiment. This choice is motivated by the fact that claims of ferroelectricity in MHPs are often invoked to explain their exceptional PV performance in terms of a fast and efficient charge separation driven by built-in electric fields, leading to low carrier recombination rates and long charge-carrier diffusion lengths. In fact, such a physical picture holds for compounds belonging to the family of ferroelectric perovskite oxides, for which BaTiO_3_ is a prominent representative. In brief, in ferroelectric semiconductors, light-to-electricity conversion proceeds through the so-called *bulk* photovoltaic effect, by which the macroscopic built-in electric field caused by the permanent ferroelectric polarization of the material leads to ubiquitous fast separation of photogenerated electrons and holes.^15,16^ Moreover, the presence of large electric fields results in the collection of hot, non-equilibrium electrons, which would enable attaining power conversion efficiencies beyond the Shockley–Queisser limit for a material with the corresponding band gap.

When considering MHPs, however, there are at least two issues that dismantle the hypothesis that their astounding PV performance is connected with ferroelectricity. First, a simple but often forgotten fact is that many high-performance perovskite materials crystallize in a **cubic** phase at room temperature or at the solar-cell working temperature. Ferroelectricity is then forbidden by symmetry: without spontaneous breaking of the symmetry, there is no deformation, and hence no possible permanent polarization. In this regard, we note that MAPbX_3_, with X = I, Br, and Cl,^17^ mixed organic-cation lead iodides FA_*x*_MA_1–*x*_PbI_3_, with *x* ∈ [0, 1],^18,19^ and lead bromides APbBr_3_, with A = Cs, MA, or FA,^20^ represent families of MHPs with similar PV performances but with room-temperature phases featuring different symmetry (cubic, tetragonal, or even orthorhombic).

A second key issue is related to the fact that alleged evidence for ferroelectricity comes almost exclusively from experiments performed in the dark, i.e., a condition completely different from that of continuous photoexcitation of a working solar cell. Furthermore, it is now well-established that photogenerated carriers in MHPs immediately form large polarons after excitation^21−24^ and that the charge transport in MHPs is dominated by polaronic effects.^25,26^ Essentially, the intrinsic lattice deformation inherent to the polaron would locally undo any octahedral deformation associated with a presumable electrical polarization.^27^ In this sense, a *polaron model*(28,29) can actually account for both the low carrier recombination rates and the surprisingly low carrier mobilities in terms of charge localization, polaron stabilization, and hopping, without the need to invoke ferroelectricity.

**Figure 1:**
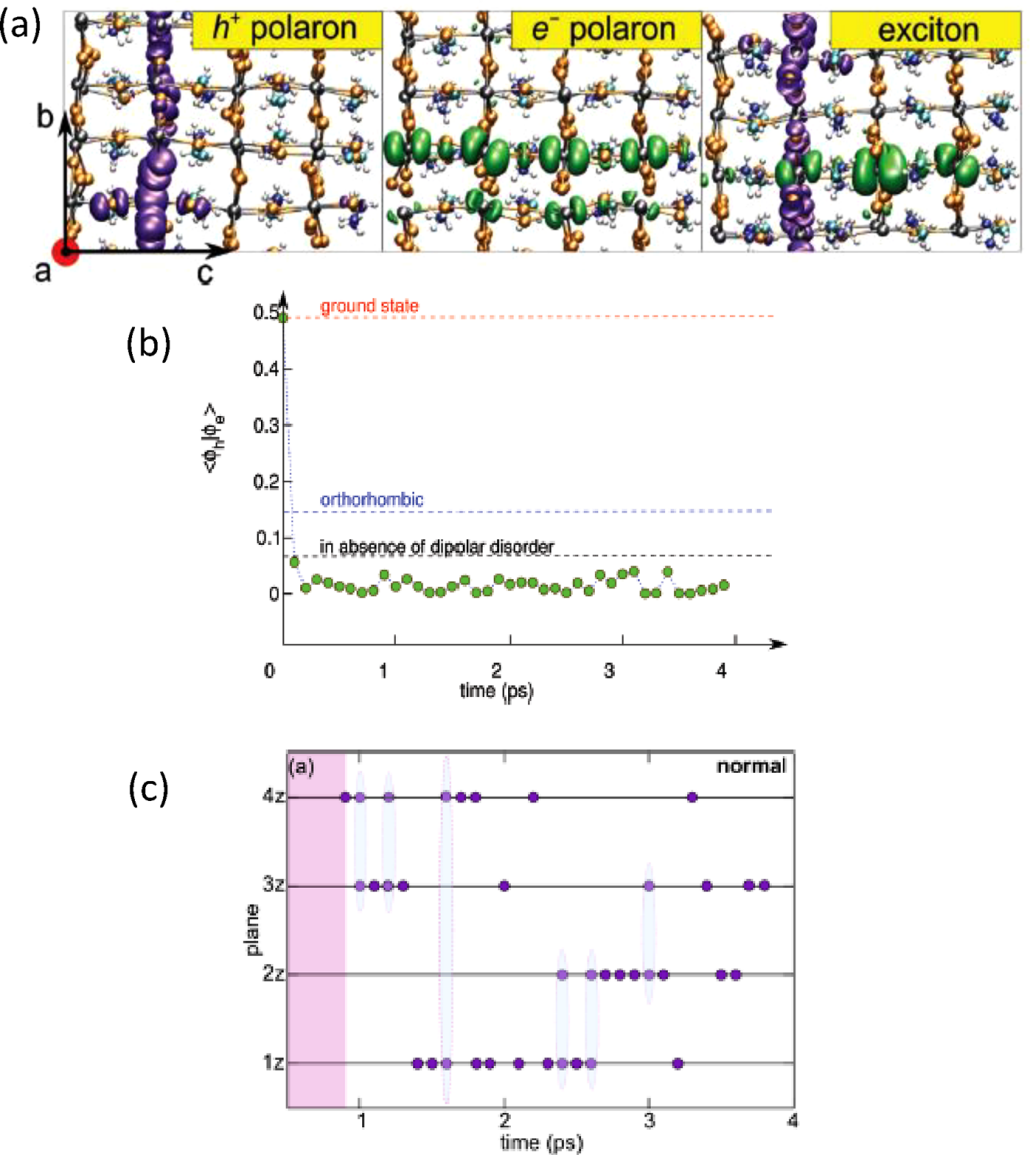
(a) Representation of the electronic isodensity of a hole polaron (left panel), an electron polaron (center panel), and an exciton (right panel) for the tetragonal phase of MAPbI_3_. Axes are indicated. Reproduced with permission from ref (22). Copyright 2019 Wiley. (b) Electron–hole wave function overlap as a function of simulation time for the exciton in the tetragonal phase of MAPbI_3_. The dashed red line represents the thermal average for the neutral exciton ground state, whereas the dashed blue line corresponds to the exciton in the orthorhombic phase. The black dashed line corresponds to an artificial system, in which the MA cations were replaced by immobile Cs^+^ ions at the inorganic cage centers. Reproduced with permission from ref (28). Copyright 2018 Royal Society of Chemistry. (c) Hopping of the charge localization during the molecular dynamics of an extra hole in tetragonal MAPbI_3_. The hole localization occurs in planes of the inorganic cage lattice perpendicular to the tetragonal axis (labeled 1z to 4z). The cyan ellipses highlight configurations in which the hole is shared among adjacent planes. Reproduced with permission from ref (29). Copyright 2019 American Chemical Society.

Ab initio molecular dynamics (MD) calculations^22,28,29^ predict that any time a free charge (electron or hole) appears in MHPs, through either photoexcitation or electrical injection, a polaron will form due to the soft nature of the polar inorganic-cage lattice. The phonons *dressing* the charge carrier are associated with stretching and bending vibrations of the inorganic cage.^30^ The polaron formation times are in the sub-ps range (0.3–0.7 ps); thus, the rotations (reorientations) of the organic cations are not directly involved in this process, because they are just too slow (ca. 3 ps).^31^ Moreover, polarons in MHPs are considered large, since the lattice distortion typically spreads over many unit cells (3–5 nm range). Their most striking characteristic is, however, that charges localize in regions which are spatially different for electrons and holes. Figure 1a displays the spatial distribution of the wave functions for isolated electrons and holes as well as for excitons (correlated electron–hole pairs) calculated for the tetragonal phase of the archetypal hybrid perovskite MAPbI_3_. A careful analysis of the wave functions reveals that holes preferentially localize on a Pb–I plane upon contraction of the Pb–I bonds, in accord with the antibonding character of the hybridized Pb(6s) and I(5p) orbitals of the top of the valence band. In contrast, electrons localize mainly along chains of elongated Pb–I bonds as a result of the antibonding Pb(6p) orbital character of the states at the conduction band minimum.^32−34^ Such spatially separated localization of electrons and holes leads to a strong reduction of their wave function overlap, which decreases by 2 orders of magnitude during polaron formation, as shown in Figure 1b for tetragonal MAPbI_3_. This effect is at the origin of the strong suppression of the bimolecular recombination in MHPs.

The previous static picture^28^ explains well the observed low charge-carrier recombination rates and long lifetimes, but, in order to address the low mobilities (see ref (6), for instance), switching to a dynamic vision within MD simulations is mandatory.^22,29^ The picture that emerges from the simulations is that polaron-assisted charge-carrier transport in MHPs has strong similarities with a hopping kind of charge transport like in organic semiconductors rather than inorganic ones. While the charge localization is induced by the dipole field generated by the organic cations, rearrangements in the inorganic sublattice provide the energetic stabilization to form polarons. Then, the polaron can rapidly hop at the sub-ps time scale between neighboring minima via a semi-localized transition state entailing a small energy barrier (150 and 80 meV for hole and electron polarons, respectively). Figure 1c illustrates the hopping process of an extra hole in tetragonal MAPbI_3_, as obtained from MD simulations. It turns out that such a jump-like movement of the polarons in MHPs arises mainly from the random reorientation of the organic cations. In fact, the motion of the A-site cation at room temperature is only marginally affected by the presence of a semi-localized charge nearby, even at the high concentration of holes considered in the simulation.^28,29^ Furthermore, this result is in accord with the observed correlation between diffusion lengths and rotation times of cations, as faster cations enable a more rapid change of the dipole field associated with the hopping.^35^ We point out that this hopping type of polaron transport also holds for fully inorganic MHPs like CsPbI_3_, where the jump-like movement of the localized charges is driven by the erratic movement of the inorganic cations between equivalent positions inside the cage voids rather than the reorientation of molecular dipoles.^36,37^

## But the Question about MHPs Being Ferroelectric Remains

A ferroelectric material is the one showing a *spontaneous and switchable* macroscopic electric polarization below a certain temperature, called the Curie temperature, in analogy to ferromagnetic ones. This definition, however, disregards the microscopic origin of the polarization. A material becomes ferroelectric typically by a displacive type of transition, in which an ion in the unit cell is displaced from its equilibrium position, when the net force from the local crystal field overcomes the elastic restoring force. As a consequence, an asymmetry in the ionic charge distribution arises, leading to a permanent dipole moment. This is certainly a necessary but not sufficient condition for ferroelectricity. In fact, of crucial importance is the occurrence of a *coupling between the permanent dipoles*, favoring their parallel alignment below the Curie temperature. Since the electric dipoles are tightly linked to the lattice, anything influencing the latter will also affect the coupling and, thus, the ferroelectric polarization. As discussed in detail below, this is where dynamic disorder in MHPs comes into play by acting directly upon the coupling between permanent dipoles, suppressing the development of a macroscopic electric polarization. We point out that in MHPs there are two other possible sources of ferroelectric order, namely the orientational polarization of the molecular cation dipoles and the ionic polarization induced by the shift of the positive charge center of A-site cations relative to the negative charge center of the PbX_3_ cage.^38^ However, at room temperature, where the A-site cation dynamics is totally unfolded, these mechanisms play no role in a macroscopic ferroelectric polarization.

Regarding the formation of permanent dipoles, however, a recent review suggests that the stereochemical expression of electron lone pairs in MHPs can be at its origin.^14^ In brief, it is concluded that, although the lone pair expression can lead to a lattice instability, breaking inversion symmetry, and thus to ferroelectricity, this effect is extremely weak in the 3D perovskites commonly used in solar cells. As a matter of fact, the octahedral distortion in terms of bond lengths and bond angles, which is caused by the repulsion between the lone pair of the central atom of the octahedrons and the valence electrons, is found to be negligible (at least 50 times lower than for true ferroelectric materials^14^), even when calculated for the less symmetric low-temperature phases of iodide and bromide compounds. Such an absence of a static polarization is consistent with a first estimate from density functional theory combined with symmetry mode analysis, where some level of entanglement was anticipated for MA-cation contributions and the role of the inorganic framework.^39^ The polarization in the tetragonal phase at low temperature, but above the phase transition to the orthorhombic phase, was found to be ca. 4.4 μC/cm^2^, which is substantially lower than for traditional perovskite oxides.

In this framework, an alternative interpretation of the properties of MHPs in terms of ferroelasticity is gaining increasing overall consensus. In this case, the material can show a spontaneous and reversible strain distribution, which is typically stress-driven and occurs at a phase transition. A ferroelastic material is prone to develop well-defined stripe patterns and/or break up into perfectly crystalline domains with alternating crystal orientation so as to minimize the macroscopic strain induced at the phase transition. As an example, we show in the Supporting Information two videos taken with an optical microscope (20× magnification) and depicting the morphological changes occurring at the surface of a big MAPbBr_3_ single crystal when the material is driven across the tetragonal-to-orthorhombic phase transition. With decreasing temperature (video-1), a set of stripes becomes clearly apparent at the phase transition. The stripes, which correspond to ferroelastic domains with alternating in- and out-of-plane *c*-axes, disappear as soon as the temperature increases beyond the transition temperature (video-2). The debate between ferroelectricity and ferroelasticity has been recently approached from a crystallographic perspective;^40^ taking MAPbI_3_ as an example, both phenomena are predicted to be possible and not mutually exclusive. However, it is inferred that, in the tetragonal *I*4/*mcm* phase, the deformation leading to the necessary broken mirror-plane symmetry would be extremely small, so as to be refined with sufficient accuracy from high-resolution single-crystal X-ray diffraction data. The opposite is true for ferroelastic domains. Although the abundant evidence in favor of ferroelasticity will be discussed in detail below, here we can anticipate that a thorough account of experimental as well as theoretical results regarding the dielectric and ferroic properties of MHPs has led the authors of ref (41) to the conclusion that MHPs might be ferroelastic electrets rather than ferroelectric. Electrets are dielectric materials presenting a combination of quasi-permanent surface and bulk charge related to real charges and/or dipoles, and the polar behavior of MHPs shows strong similarities with that of electrets.

## Results Interpreted as Evidence of Ferroelectricity

**Figure 2:**
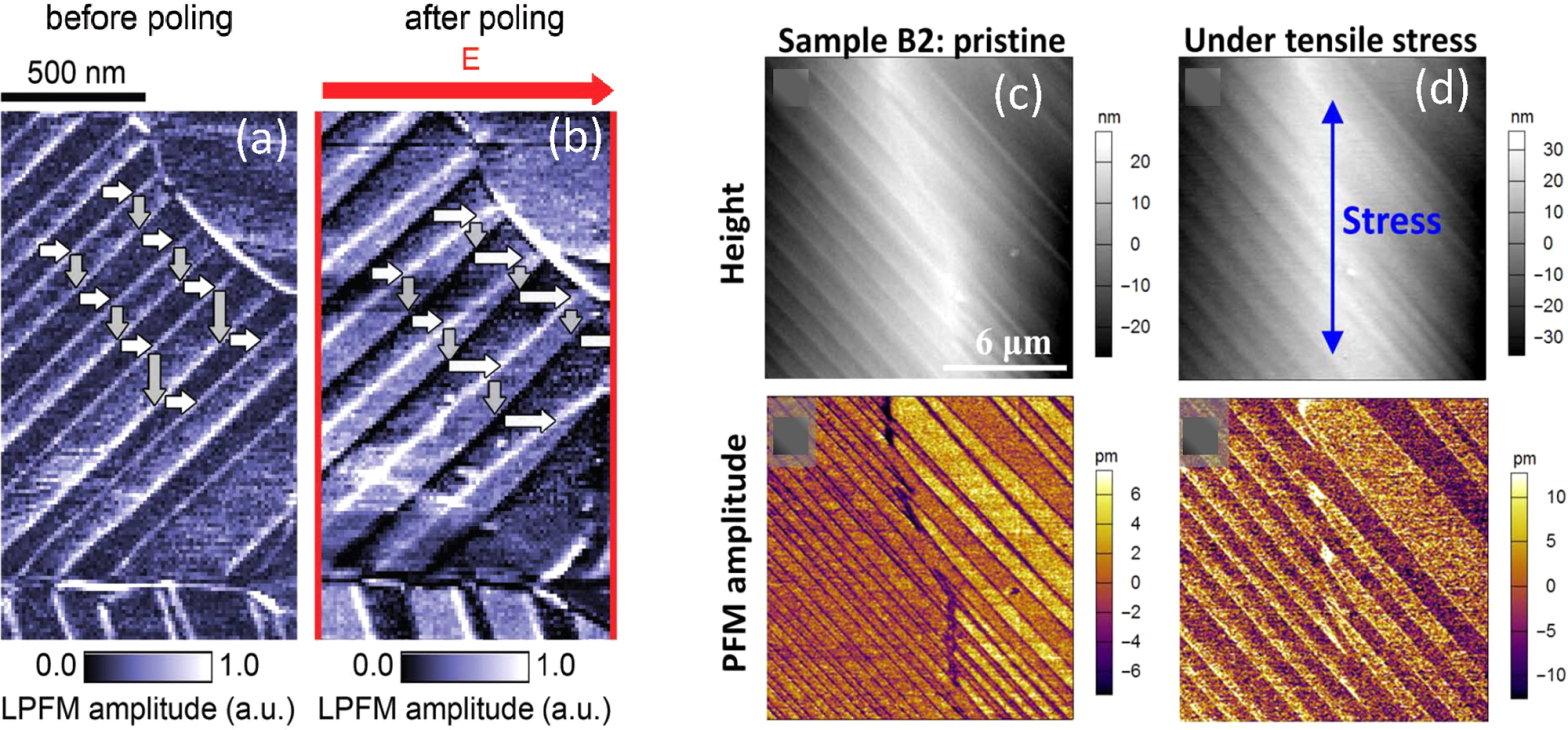
Observation of domains interpreted as ferroelectric. Lateral-PFM amplitude of a MAPbI_3_ thin film (a) before and (b) after poling with an in-plane field (red arrow) of *E* = +4.5 V μm^–1^ for 11 min, illustrating how the size and shape of domains change significantly upon the application of the electric field. The white/gray arrows’ lengths represent the projected width of each domain in the polarization direction. Reproduced with permission from ref (47). Copyright 2019 Wiley. Observation of ferroelastic domain patterns modulated by external stress. Topography and PFM amplitude images of a MAPbI_3_ film fabricated by doctor blade-coating (c) in the pristine state and (d) under tensile stress. The blue arrow indicates the direction of the applied stress. Reproduced with permission from ref (52). Copyright 2017 American Association for the Advancement of Science.

There is a long series of experimental as well as theoretical results interpreted as evidence for the existence of both a ferroelectric polarization and ferroelastic domains. A very nice chronology of the milestones is provided in a recent review from 2021 (ref (42)—see references therein). However, the strongest claims in favor of ferroelectric behavior arise from the observation of domains using different kinds of piezo-response force microscopy (PFM) in either thin films^43,44^ or single crystals.^45^ One of the main observations involves lateral-PFM images of domains containing series of (bright/dark) stripes (see ref (46), for instance). The stripe pattern is interpreted as being due to ferroelectric domains with in-plane polarization, pointing at 90° from stripe to stripe (see Figure 2a). The contrast in lateral PFM may arise from the alternating orientation (parallel or perpendicular) of the presumably ferroelectric polarization with respect to the cantilever axis of the scanner. Moreover, the poling of domains with electric fields is widely taken as the ultimate evidence of ferroelectric behavior.^45,46^ For that purpose, Röhm et al.^47^ have developed a poling setup based on a pair of interdigitated finger electrodes deposited on top of a MAPbI_3_ film. Although they observed a clear development of well-defined sets of fringes that can act upon the stripes by applying a lateral (in-plane) DC electric field, as shown in Figure 2a,b, we anticipate a possible reinterpretation of these results in terms of ferroelasticity. Furthermore, even among the supporters of the existence of ferroelectricity in MHPs, the nature of the ferroelectric polarization is not completely clear. Recently, it has been claimed that the contrast captured in the PFM experiments corresponds to the coexistence of polar (ferro) and nonpolar (antiferro) domains.^48^ Arguments are based on structural broken symmetries and statistical synthesis. However, the A-site cation dynamic disorder is totally disregarded. Finally, by analyzing the results from a variety of different characterization techniques, Garten et al.^45^ were led to the conclusion that MHPs exhibit *relaxor* ferroelectric behavior. In this case, the ferroelectric polarization breaks up into nanopolar regions, such that the material can mimic either a ferroelectric or a dipolar glass, depending on external conditions regarding electric fields and temperature. In this way, it is claimed that the discrepancies between different reports can be explained. We will show below that dynamic disorder plays against ferroelectric order even at the nanoscale.

## Results Pointing to Ferroelastic Behavior

**Figure 3:**
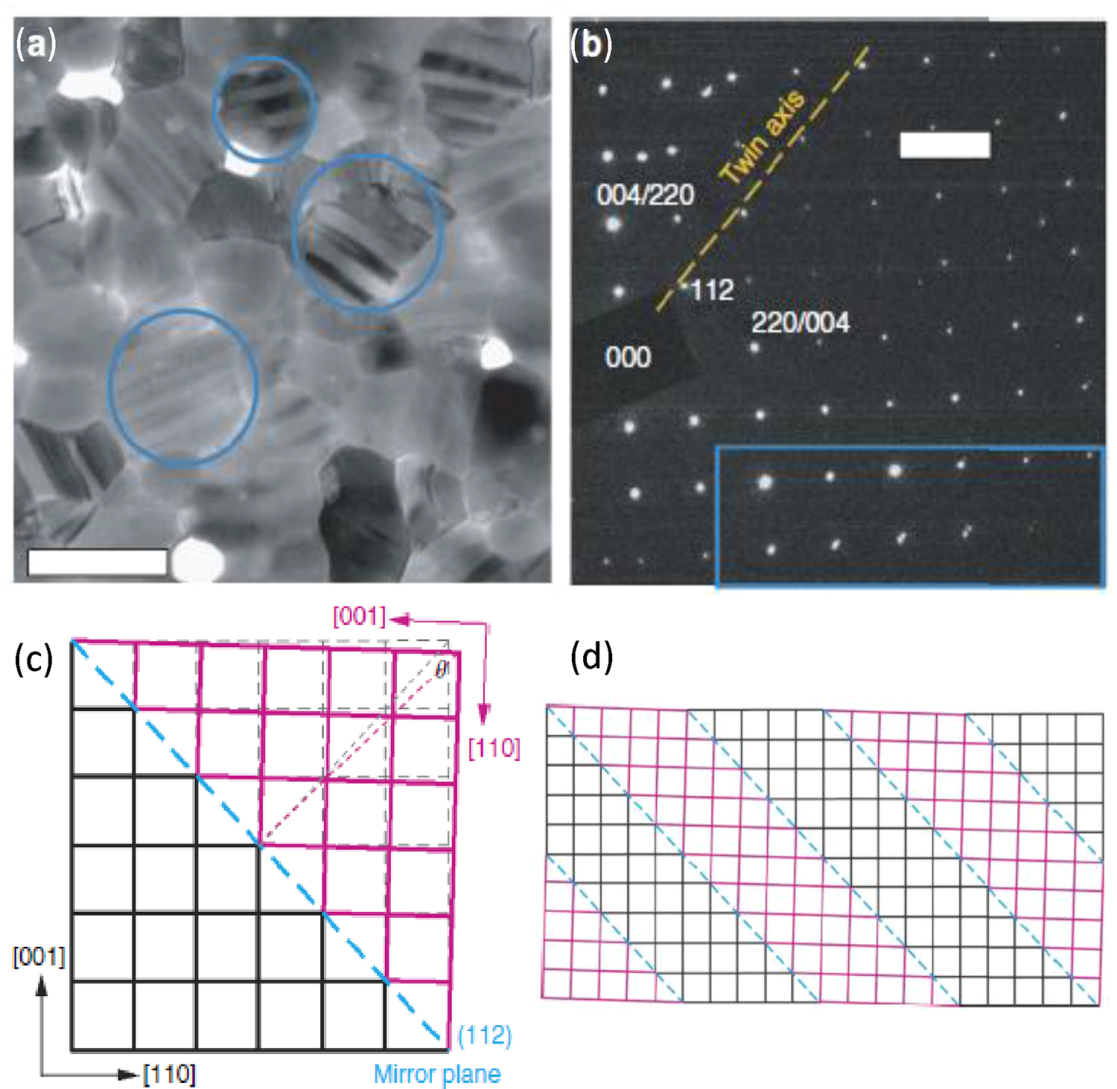
Observation of ferroelastic domains in electron-beam transmission and diffraction measurements. (a) Low-dose, bright-field transmission electron microscopy (TEM) image of a pristine MAPbI_3_ thin film at room temperature. A stripe contrast is visible through some of the grains (examples circled in blue). (b) Diffraction pattern taken from a grain exhibiting stripe contrast, showing two single-crystal patterns with a mirrored relationship. (c) Schematic of the proposed twinning geometry of the MAPbI_3_ lattice. The original lattice without twinning is shown in dashed lines. (d) Schematic of the proposed twin-domain structure regarding the observed stripes. All indexes are for the tetragonal phase. Reproduced with permission from ref (50). Copyright 2017 Springer Nature.

The experimental evidence in favor of a ferroelastic behavior of the perovskites, although shown mostly in MAPbI_3_, is robust in the sense that the observation of stripes is usually accompanied by structural information gained by X-ray diffraction,^49^ electron-beam diffraction,^50,51^ or a combination of PFM and photothermal-induced resonance,^52^ among others. Figure 2c,d provides an example of the switching of the ferroelastic domains when an external stress is locally applied by increasing the force of the PFM tip (<40 nN) exerted on the sample during the scan in contact mode.^52^ The structural information is crucial because it serves to demonstrate that the stripes indeed correspond to ferroelastic twin domains of the tetragonal phase, with the in-plane [001] (long) and [110] (short) crystal axes in one domain rotated 90° with respect to the other domain, for example, as depicted in Figure 3. The stripes’ boundaries are determined by the domain walls which run along (112) mirror planes.^50^ The formation of twin domains is simply triggered by a ubiquitous mechanism of strain compensation, when the material transforms from the cubic to the tetragonal structure. Both the elastic properties and the kinetics of the phase transformation determine the size (width and length) of the domains, as well as the interaction with the substrate for the case of supported thin films.

## Can the Supposed Experimental Evidence for Ferroelectricity Be Interpreted in Another Way?

Any ferroelectric material is piezoelectric, but the reverse is not true. An external voltage applied to any piezoelectric material produces a deformation (strain) proportional to the external electric field; conversely, the application of a force (stress) induces a polarization charge proportional to the strain. In this respect, PFM measurements always yield the material’s piezoelectric coefficients *d*_μν_, which correspond to specific piezoelectric tensor components, depending on sample geometry and measurement configuration. Thus, a ferroelectric polarization cannot be directly assessed by scanning probe microscopy techniques, which means that the mere observation of domains is not necessarily evidence of ferroelectricity. In the case of MHPs and specifically MAPbI_3_, the demonstration of ferroelectricity might be hopeless, because of a faint ferroelectric polarization^53^ with an upper bound determined at 1 μC/cm^2^ ^54^ and the extremely weak electromechanical response revealed in experiments.^42,55^ Hence, taking for granted that most of the experimental results are free of artifacts, it is plausible that the domains observed in MHPs by PFM and claimed to be ferroelectric can be at last explained by invoking ferroelastic behavior, as already suggested.^41,56,57^ The situation is similar when we consider the poling of the domains, demonstrated using interdigitated finger electrodes.^47^ We point out that such electrodes are used to generate surface acoustic waves (SAWs), even in weakly piezoelectric materials like GaAs.^58^ SAWs are low-frequency acoustic phonons with a linear energy–wave–vector dispersion, which collectively produce dynamic strain fields propagating along the surface of the solid. A crucial issue is that the interdigitated fingers shown in ref (47) generate a *static* strain field, having much larger inter-finger distances up to 10 μm. When a DC bias is applied to the interdigitated finger electrodes, the piezoelectric perovskite film becomes periodically strained, exhibiting alternating regions of compressive and tensile strain commensurate with the finger array. For such large inter-finger distances, strain accumulation just triggers the formation of ferroelastic twin domains in order to release elastic energy, minimizing the average macroscopic strain in the film. A poling of the bias causes the reorganization of the domains as observed in PFM. In addition, the lateral field of the interdigitated fingers would also lead to ion migration,^59^ inducing further lattice strain from alternating ion accumulation close to the electrodes.

In addition, the dielectric behavior of MHPs at very low frequencies, in the MHz range, has been taken as evidence for ferroelectricity^60^ or relaxor ferroelectricity.^45^ However, no indication of ferroelectric ordering was found for the three methylammonium lead halide compounds (MAPbX_3_, with X = Cl, Br, I) from frequency- and temperature-dependent dielectric measurements across the entire frequency spectrum, despite the fact that the dielectric constant conserves very high values (>27) for frequencies below 1 THz in all three halides.^61^ In fact, the steep increase in the low-frequency dielectric response of tetragonal MAPbI_3_ below 3 cm^–1^ can be quantitatively explained in terms of different contributions (lattice, molecular, and displacement contributions) as due to the onset of rotational modes of the molecular cations.^62^ Simulations show that the dominant contribution arises from the coupling between inorganic-cage lattice vibrations and librational modes of the molecular cations. This result was obtained under conditions of an on-the-average apolar structure as a consequence of the fast molecular cation dynamics, clearly indicating that ferroelectricity is not necessary to explain the dielectric behavior of the perovskites.^62^

Hysteresis in the current–voltage characteristics of MHP solar cells^63^ as well as in PFM experiments^45,64^ was also taken as evidence of ferroelectricity. In contrast, several reports indicated that such a behavior was related to ion migration (mainly the halides), favored under the combined influence of the built-in electric fields and light.^54,65,66^ Ionic motion was also invoked to explain the much slower decay of the current (in the range of 1 s) in variable force PFM experiments in a trication halide perovskite film.^53^ For completeness, we refer to a thorough account of ionic transport and the associated defect chemistry in hybrid halide perovskite solar cells published recently.^67^

## A Unique Structure/Organic-Cation-Dynamics Relationship

**Figure 4:**
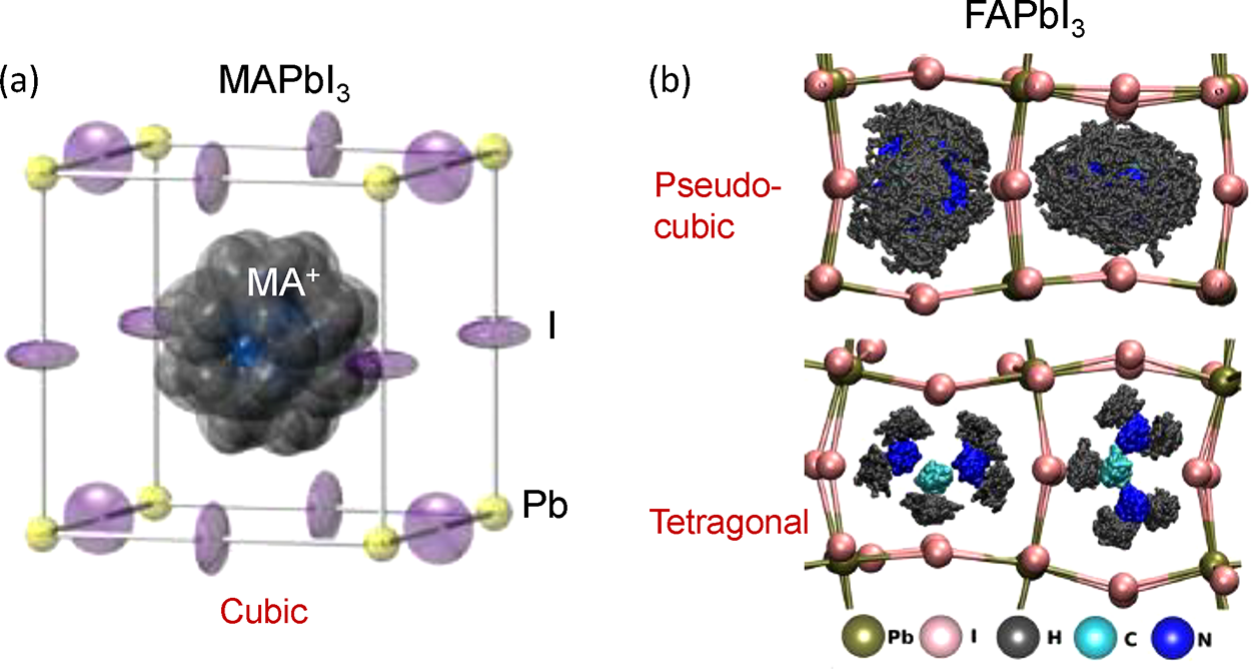
(a) Orientational disorder modeled in the cubic phase of MAPbI_3_ at 352 K, given by the atomic displacement parameter (ADP) ellipsoids at 50% probability obtained from neutron powder diffraction measurements. Atom key as follows: lead yellow, iodine purple, carbon black, nitrogen blue, and hydrogen gray. Reproduced with permission from ref (68). Copyright 2015 Royal Society of Chemistry. (b) Trajectory of FA cations inside the A-site cages for the pseudocubic and tetragonal phases of FAPbI_3_ from molecular dynamics simulations. The green and pink spheres represent the time-averaged positions of Pb and I, respectively. Reproduced with permission from ref (69). Copyright 2018 American Chemical Society.

A salient feature of MHPs is the unique relationship between the structural properties of the soft lattice of corner-sharing metal halide octahedra and the A-site cation dynamics. The latter is unfolded in the cubic and tetragonal phases but totally locked in the less symmetric orthorhombic phase. Such an organic cation dynamics is a well-established phenomenon that builds upon the results, for example, of ultra-fast pump and probe spectroscopy, where typical molecular libration and rotation times from 0.3 to 3 ps were determined.^31^ Concomitant with the A-site cation dynamics, a 3D atomic probability cloud was inferred for the organic molecules, while they are freely rotating inside the inorganic cage voids, as determined from neutron scattering experiments for MAPbI_3_^68^ and from MD simulation in FAPbI_3_,^69^ as illustrated in Figure 4. We emphasize that the dynamic steric interaction between the halogen anions and these A-site cation probability clouds is instrumental for the structural stabilization of the different phases of the MHPs.^70,71^

Several experimental results point to a one-to-one correlation between the degrees of freedom of the inorganic cage and the molecular cations. One of the most direct ones corresponds to the prominent effect that the locking of the organic molecules within the inorganic cage has on the line widths of the phonon modes of the inorganic lattice, as observed in temperature-dependent Raman spectra.^72,73^ For example, in the cubic and tetragonal phases of MAPbI_3_, the low-frequency Raman peaks below ca. 200 cm^–1^, corresponding to the inorganic cage phonons, are extremely broad and poorly resolved. This is a direct consequence of the dynamic disorder caused by the fast librations and rotations of the MA cations. Coinciding with the temperature-induced phase transition into the orthorhombic phase at 162 K, a marked reduction of the Raman line widths by a factor of 5–10 is clearly observed in Raman scattering experiments. This is due to the disappearance of the dynamic disorder, as the A-site cations become locked inside the contracted and less symmetric cage voids of the orthorhombic phase.^72,73^

Compelling evidence for the tight structure/MA–dynamics relationship, however, is provided by the combination of PL and Raman experiments under high pressure at ambient temperature.^74^ At a very low pressure of ca. 0.4 GPa, MAPbI_3_ transforms from the tetragonal phase, stable at ambient conditions, into a *cubic* structure, as clearly demonstrated by single-crystal X-ray diffraction.^75^ The occurrence of this phase transition is corroborated by the high-pressure PL and Raman experiments, the latter still indicating a fully unlocked dynamics of the MA molecules, as judged from the large Raman line widths. Such a structural transformation path from tetragonal to cubic under pressure is very unusual, being in stark contrast with the transformation into a less symmetric orthorhombic structure that takes place when the temperature is decreased at ambient pressure. The explanation for this phenomenon is found in the results of MD simulations performed as a function of hydrostatic pressure for FAPbI_3_ and a mixed-cation compound, FA_1–*x*_Cs_*x*_PbI_3_.^71^ The pressure-induced structural distortions have a major influence on the FA cation dynamics, and vice versa. Under compression, stronger hydrogen bonds result in enhanced dynamical coupling between the FA cations and the inorganic Pb/I sublattice. The fast reorientation of the molecular axis due to jump-like rotations of the FA cations inside the cage voids leads to an atomic probability cloud with spherical symmetry. This, in turn, favors a cubic environment, that is, a cubic arrangement of the corner-sharing PbI_6_ octahedra.

## Role of Organic-Cation Dynamics in the Ferroic Properties of Perovskites

An important corollary of the MD simulations concerns the local distortions induced in the Pb/I sublattice due to the jump-like rotations of the organic cations. The dynamical steric interaction between the hydrogen atoms of the organic molecules and the halide atoms of the inorganic sublattice leads to local changes in the metal halide bond lengths and angles, distorting the octahedra. This is the reason for the pronounced broadening of the Raman peaks associated with the inorganic cage phonons, when the molecular cation dynamics is unfolded. Needless to say, these dynamic distortions will destructively impact any possible ferroelectric order associated with the polarization stemming from the stereochemical expression of the Pb lone pairs. For the system of electric dipoles, the organic cation dynamics mimics a thermal bath with an effective temperature above the ferroelectric Curie temperature. In a sense, the perovskite would behave more like a paraelectric material rather than ferroelectric.

For the low-temperature orthorhombic phase, even though the organic cations are locked in the voids of the inorganic cage, a simple paraelectric behavior is observed.^76^ In fact, the locking of the cations impairs their reorientation upon application of an external electric field, such that the paraelectric response of the material comes solely from the polarization of the inorganic cage. At last, it is the symmetry of the orthorhombic phase which does not allow ferroelectricity, regardless of the organic cation’s orientation.^77^ In addition, we point out that a higher bimolecular recombination rate has been measured, e.g., for the orthorhombic phase of MAPbI_3_,^9^ which has been explained in terms of a higher degree of delocalization and hence a higher overlap for holes and electrons, when compared to the more efficient tetragonal phase.^28^ This further corroborates the lack of correlation between the supposed ferroelectricity and the outstanding performance of MHPs.

At variance with this, the cubic and tetragonal structures of the metal halide sublattice are fully compatible with the 3D and 2D rotational dynamics of the organic cations, respectively. As a consequence, ferroelastic order is solely driven by the macroscopic build-up of strain but is not affected by the cation dynamics. Moreover, there is clear evidence that ferroelastic domain formation has a benign influence on charge-carrier transport, for example, enhancing the photoelectric response in CsPbBr_3_^78^ or inducing an anisotropy in the carrier diffusion in MAPbI_3_ due to the presence of twin domains.^79^ In fact, domain walls have been proposed to play a crucial role in the extremely long lifetimes of photogenerated charge carriers in MHPs. An interfacial polaron formation model, developed to account for the long diffusion lengths extracted from photoinduced absorption measurements in MAPbI_3_,^80^ indicates that positively and negatively charged polarons are readily trapped at domain walls but at different places along the interfaces, according to particular lattice deformations, further reducing charge-carrier recombination and extending their lifetime.

In conclusion, we aimed at settling the dispute regarding the existence/absence of ferroelectric domains in MHPs and their impact on the astounding electronic properties of this class of materials. To this end, we critically discussed the main experimental evidence in favor and against ferroelectricity, also in view of the physical picture arising from theoretical modeling. Our analysis points out the key factors demonstrating that, under operative conditions of a solar cell, ferroelectric domains in MHPs neither can be sustained nor should be relevant for the physics of charge carriers. We proposed an alternative interpretation of the measurements, based on the concept of ferroelasticity, which can reconcile contradictory findings and indeed is proved to have a clear impact on the charge transport in MHPs.

## Supporting Information Available

Observation under optical microscope of the tetragonal-to-orthorhombic phase transition, occurring at 152 K, by cooling a MAPbBr_3_ single crystal at approximately 20 K/min (MP4)
Observation under optical microscope of the orthorhombic-to-tetragonal phase transition, occurring at 152 K, by heating a MAPbBr_3_ single crystal at approximately 20 K/min (MP4)
